# Effect of Social Service Prenatal Care Utilization on Perinatal Outcomes among Women with Socioeconomic Problems in the Tokyo Metropolitan Area

**DOI:** 10.5402/2011/856027

**Published:** 2011-10-25

**Authors:** Jun Kakogawa, Miyuki Sadatsuki, Yoko Ogaki, Misao Nakanishi, Shigeki Minoura

**Affiliations:** Department of Obstetrics and Gynecology, National Center for Global Health and Medicine, 1-21-1, Toyama, Shinjuku-ku, Tokyo 162-8655, Japan

## Abstract

*Background*. To investigate the effect of social service prenatal care (PNC) utilization on perinatal outcomes among women with socioeconomic problems in the Tokyo metropolitan area. *Methods*. Retrospective study. The study enrolled all women at our hospital who either attended PNC utilizing social services (attenders) or who did not attend PNC (nonattenders) between January 1, 2007, and December 31, 2010. We compared the maternal characteristics and perinatal outcome of attenders with those of nonattenders. *Results*. A total of 83 attenders and 45 nonattenders were enrolled. The mean gestational age at the first PNC visit was 31.1 weeks in the attenders. Attenders were found to have a lower incidence of preterm delivery, pregnancy-induced hypertension, emergency cesarean section, low birth weight, and the NICU admission than nonattenders (*P* < 0.05). *Conclusions*. The utilization of social service PNC greatly improved perinatal outcomes among women with socioeconomic problems problems in the Tokyo metropolitan area.

## 1. Introduction

Prenatal care (PNC) is a frequently used health service that has the potential to reduce the incidence of perinatal morbidity and mortality. However, the effectiveness of PNC remains equivocal [1, 2], and high-quality evidence is scarce [3, 4]. The Japan Society of Obstetrics and Gynecology (JSOG) [5] recommends approximately 14 PNC visits, with the first visit to be held prior to 11 weeks of gestation. A lower number of PNC visits may be adequate for low-risk pregnancies if high-quality care can be provided and if problems are detected promptly and properly addressed [6, 7]. Randomized controlled trails suggest that fewer PNC visits can be as effective as standard models of PNC for low-risk women and are not associated with different pregnancy outcomes [4, 8].

Several studies have reported that inadequate PNC carries a substantially elevated risk of severe adverse prenatal outcomes [9, 10]. Previous studies have investigated the rates of inadequate PNC: 1.7% in Kuopio, Finland [9], 8.3% in Winnipeg, Monitoba, Canada [11], and 33.9% in Aracaju, Brazil [12]. In Japan, there is no national data regarding inadequate PNC; however, the incidence of women without PNC in Japan has been estimated at approximately 0.3% [13], compared to about 1.5% to 2.0% of pregnant women in the United states who do not receive any PNC [14]. Currently in Japan, advances in clinical practice, improved socioeconomic status, and financial support from public funds for PNC contribute to the low maternal mortality (4.8 per 100,000 births) and perinatal mortality (4.3 per 1,000 births) [15]. Regardless of high-quality clinical practice and readily accessible PNC in Japan, poor birth outcomes among the minority of pregnant women who do not receive PNC continues to be a serious problem.

It has been reported that there are socioeconomic barriers to PNC (i.e., low income, low education level, unmarried status, and teenage pregnancy) [16]. Health and social services can help improve pregnancy outcomes [17]. There exist some social services for pregnancies and child deliveries in Japan. One of the services is the Women's Protection Facilities (WPFs), managed by the Women's Consulting Offices in each prefecture. WPFs provide accommodation and meals for women with socioeconomic problems, where they can safely live for short periods of time. Some pregnant women stay in WPFs during their pregnancies and receive PNC and deliver their infants at hospitals.

Although it is considered important to promote access to social services for women with socioeconomic problems, little attention has been focused on assessing the efficacy of prenatal social service utilization on perinatal outcomes among women with socioeconomic problems. This may be due to the limited number of people affected. Thus, the purpose of this study was to investigate the effect of social service prenatal care utilization on perinatal outcomes at the National Center for Global Health and Medicine (NCGM) in the Metropolitan Area of Tokyo among women with socioeconomic problems.

## 2. Materials and Methods

The study enrolled all women who stayed in WPFs and attended PNC utilizing social services (attenders) or who did not attend PNC before their deliveries (nonattenders) at the National Center for Global Health and Medicine (NCGM) between January 1, 2007, and December 31, 2010. Exclusion criteria included multiple pregnancy (two cases in the attenders group). The study was approved by the institutional review board of NCGM.

Data were retrospectively retrieved from a perinatal database followed by an individual chart review. Outcomes we investigated included the reasons for not attending the PNC workshops, the reasons for staying at a WPF, maternal characteristics concerning marital status, the relationship with the child's father, obstetric history, maternal medical complications, obstetric outcomes, and neonatal morbidity. We compared the maternal characteristics and perinatal outcome of attenders with those of nonattenders.

NCGM is the only public hospital in Shinjuku (the Metropolitan Area of Tokyo) equipped with an NICU, where medical care is available to the poor population of Shinjuku and the surrounding areas. Most women with socioeconomic problems who reside in this area deliver their infants at NCGM, and women in this area who do not attend PNC workshops at any medical institution and call for an ambulance after the onset of labor are transported to NCGM. Furthermore, only one WPF is managed for pregnant women in Tokyo. NCGM is located ten minutes by car from the WPF for pregnant women. Therefore, most pregnant women who stay in the WPF in Tokyo deliver at NCGM. 

The following definitions were used. Young maternal age was defined as age less than 20 years. Gestational age was estimated based on the first day of the last menstrual period. Single status was defined as any civilian status other than marriage (including cohabiting, single, widowed, and divorced women). Cigarette smoking and alcohol consumption during pregnancy were recorded on a perinatal database as yes/no. Pregnancy-induced hypertension (PIH) was defined as a blood pressure of 140/90 mm Hg or higher after the 20th week of gestation. We defined preterm delivery as a gestational age less than 37 weeks. Low birth weight (LBW) was defined as less than 2,500 g; birth weight greater than 4,000 g was defined as macrosomia. Stillbirth was defined as an intrauterine death of a fetus at 22 or more weeks of gestation. Early neonatal death was defined as death of a neonate during the first seven days of life; late neonatal death was defined as death between eight and 28 days after birth. The admission rate to the neonatal intensive care unit (NICU) was recorded as infants who required more than 24 hours of surveillance. 

The data are presented as means ± SD. The Statistical Package for the Social Sciences (SPSS, version 10.0 for Windows; SPSS, Inc., Chicago, Ill, USA) was used to analyze the data. Dichotomous data were compared with Chi-square tests, and Fisher's exact test was applied when the minimal estimated expected value was less than five. Continuous variables were analyzed by a Student's *t*-test. A *P* value of <0.05 (95% confidence interval) was considered statistically significant.

## 3. Results

A total of 83 attenders and 45 nonattenders were enrolled in this study. There were 2,084 deliveries at NCGM during the study period. The proportion of attenders (3.9%) and nonattenders (2.1%) among all deliveries was small during the study period. In the attenders group, the reasons for staying at a WPF included poverty in 67 cases (80.7%), victims of domestic violence in 12 cases, and victims of rape in four cases. Of 83 attenders, 32 had no PNC before staying in the WPF and utilizing social services. Of 51 attenders who had PNC before staying in the WPF and utilizing social services, only two had regular PNC. Therefore, in the attenders group, the mean gestational age at the first PNC visit was 31.1 ± 5.4 weeks. In the nonattenders group, the reasons for not attending PNC included poverty in 37 cases (82.2%), unaware of a pregnancy in three cases, requesting divorce in two cases, fear of pregnancy and delivery in two cases, and busy with child care in one case.

Table 1 describes the demographic and maternal characteristics of the study participants. There were no differences in the mean maternal age, the incidence of young maternal age, primiparas, unmarried status, cigarette smoking, alcohol consumption or history of divorce between attenders and nonattenders.  Table 2 describes the maternal complications in the study groups. Attenders were found to have a lower incidence of PIH compared with rates of nonattenders (*P* < 0.05). Attenders were found to have a lower incidence of personality disorder compared with rates of nonattenders (*P* < 0.05).

Table 3 describes the pregnancy outcomes in the study groups. Gestational age at delivery in nonattenders was earlier than in the attenders (*P* < 0.05). Attenders were found to have a lower incidence of preterm delivery and emergency cesarean sections compared with rates of nonattenders (*P* < 0.05). In the attenders group, the indications for emergency cesarean section included two cases of fetal distress and one case of placenta previa. In the nonattenders group, the indications for emergency cesarean section included three cases of fetal distress, two cases of previous cesarean section, two cases of breech presentation, and one case of eclampsia. Attenders were found to have a higher incidence of forceps deliveries compared with the rate of nonattenders (*P* < 0.05). In the attenders group, the indications for forceps delivery included four cases of fetal distress and four cases of prolonged labor. Nonattenders were found to have a higher incidence of delivery outside of a hospital compared with those of attenders (*P* < 0.05). Of five cases delivered outside of a hospital in the nonattenders group, the place of delivery was home in two cases, ambulance in two cases, and on the street in one case. No maternal deaths occurred in either group.

Table 4 describes the neonatal characteristics and reasons for admission to the NICU in the study groups. There were no stillbirths or neonatal deaths in the study groups. Although there were no differences in the incidence of macrosomia and low Apgar score, birth weight in nonattenders was lighter than in the attenders (*P* < 0.05). Attenders were found to have a lower incidence of low birth weight and admission to the NICU compared with rates of nonattenders (*P* < 0.05). In regard to the reasons for admission to the NICU, neonates from nonattenders were found to have a higher incidence of preterm delivery, low birth weight, neonatal infection, and asphyxia (*P* < 0.05). In the nonattenders group, two cases were diagnosed with syphilis after delivery, and their neonates were diagnosed with congenital syphilis.

## 4. Discussion

In this study, we investigated the effect of social service prenatal care utilization on perinatal outcomes among women with socioeconomic problems in the metropolitan area of Tokyo. As we described, this study revealed that women who attend PNC utilizing social service had better perinatal outcomes than women who did not attend PNC despite the fact that mean gestational age at the first PNC visit was 31.1 weeks for those who attended a workshop. Specifically, the incidence of emergency cesarean section, preterm birth, admission to the NICU, neonatal infection, and asphyxia were found to be statistically lower for the attenders than for the nonattenders.

Perinatal assistance in the metropolitan area of Tokyo is characterized by easily accessible and high-quality maternity care, but there are some inadequacies that may compromise optimal outcomes. The present study demonstrated a similar profile to earlier studies. Although there were only 3.9% of attenders and 2.1% of nonattenders among all deliveries during the study period, the present study showed that socioeconomic and demographic barriers to adequate PNC utilization still exist in Tokyo. There were no differences in the demographic and maternal characteristics between attenders and nonattenders in the present study. Therefore, there is a possibility that patients who received PNC in the present study may have delivered their infants without any PNC if they were not provided the opportunity for social service PNC. 

The necessity for early access to PNC has been reported to permit identification of risk factors early in pregnancy and reduce maternal morbidity and the neonatal consequences [18]. It has been reported that low PNC attendance carries a substantially elevated risk of severe adverse perinatal outcomes [19, 20]. Although the total number of PNC visits was reduced in the attenders group in this study due to late attendance at the first PNC, attenders were found to have a lower incidence of PIH compared with rates of nonattenders. Interestingly, there was no difference in the incidence of preterm delivery (3.6%) or LBW (9.6%) in attenders when compared with the maternal and child health statistics of Japan, 2008 (preterm delivery: 5.8%, LBW: 9.6%) [15]. One reason of this improvement in perinatal outcomes for attenders was that they could attend PNC workshops and receive appropriate interventions by utilizing social services despite their late initiation of PNC. Another possible reason was that their living environment and daily nutrition were improved while residing in the WPF. We believe that the present study demonstrates the impact of social service PNC utilization on perinatal outcomes among women with socioeconomic problems. 

Several studies have demonstrated that inadequate PNC is associated with preterm delivery [9, 11], LBW [9, 11], and increased perinatal morbidity and mortality among LBW infants [8, 14, 21]. Clinically, inadequate PNC appeared to be a contributor to LBW, and this association was chiefly the result of preterm deliveries not growth restriction [11]. The incidence of preterm delivery in the nonattenders (22.2%) was six times higher than for those who utilized social services (3.6%) in the present study. This difference can be explained by the fact that nonattenders likely did not receive appropriate treatment and preventive care. Furthermore, it could explain that high incidence of preterm delivery influenced the frequency of admission to the NICU in the nonattenders. Preterm delivery remains one of the principal causes of perinatal mortality and morbidity [22]. The present study demonstrated that strategies to promote the attendance of social service PNC could decrease of the incidence of preterm deliveries. 

In the present, attenders were found to have a lower incidence of emergency cesarean sections compared with rates of nonattenders. The explanation of this results is that women who delivered by emergency cesarean sections in the nonattenders would have had planned cesarean sections if they had taken part in PNC although there were no difference in the incidence of previous cesarean section, breech presentation, and eclampsia between attenders and nonattenders when analyzed independently. Although attenders were found to have a higher of forceps deliveries compared with rates of nonattenders, it has been considered that they could succeed in vaginal deliveries by receiving PNC and adequate management of labor and delivery. 

Pregnancy is at times sufficiently stressful to provoke psychiatric disorders. Attenders were found to have a higher incidence of personality disorder compared with rates of nonattenders. There is a possibility that response to stress due to pregnancy and socioeconomic problems among women with personality disorder may be seen as anxiety developed about the lifestyle change, labor pain, and child care throughout pregnancy, especially toward term. Therefore, they could have opportunities for utilizing social service by seeking psychiatric and economical support. 

We consider the main limitation of this study is its small sample size. However, we consider that the results of the present study could represent the effect of social service prenatal care utilization on perinatal outcomes in the Metropolitan Area of Tokyo since NCGM is the only public hospital equipped with an NICU, where medical care is available to the poor population of this area and most pregnant women who stay in the WPF in Tokyo deliver. Another possible limitation is that an inaccurate ascertainment of gestational age may affect the determination of a preterm delivery or LBW infant. Because background data for nonattenders was collected at the time of delivery, underreporting could have been a source of error, depending on the pregnancy outcome. However, inaccuracies while assessing the risk of prematurity was overcome by a high percentage of low birth weight infants in the nonattenders group. Our analysis was limited to singleton pregnancies; therefore, multiple-gestational pregnancies were not represented. In addition, the type of social services utilized was limited to women staying in WPFs during their pregnancies; however, there are many kinds of social services. Therefore, further investigation should be considered to analyze the effects of prenatal care on perinatal outcome when utilizing other social services. Furthermore, we think it is necessary to investigate perinatal outcomes according to the degree of PNC attendance.

In conclusion, this study revealed that social service PNC utilization greatly improved perinatal outcomes among women with socioeconomic problems. Our findings identified the fact that socioeconomic inequalities are important factors associated with PNC attendance and adverse perinatal outcomes in the Tokyo metropolitan area. We stress that it is important to disseminate information and inform women with socioeconomic problems how to access and utilize social services in order to prevent serious maternal and neonatal health problems and to improve overall perinatal outcomes. To minimize perinatal risks for women with socioeconomic problems, intervention must begin before conception or at the early stages of pregnancy. There is a pressing need for further research to identify areas where new interventions might encourage the utilization of services and to gauge the likely impact of increased dissemination of information about the availability of social services. We plan to conduct further clinical investigations to help reduce the number of women with inadequate PNC and to promote improved pregnancy outcomes.

##  Conflict of Interests

The authors have no conflict of interests.

## Figures and Tables

**Table 1:** Demographic and maternal characteristics in the study groups. Data include the number of women presented as the mean ± standard deviation. Attenders had a statistically significantly higher rate of not having a relationship with the child's father (*P* < 0.05). Significance at *P* < 0.05 was analyzed by Fisher's exact test. NS: not significant.

	Attenders (*n* = 83)	Nonattenders (*n* = 45)	*P* value
Maternal Age (years), mean ± SD	26.2 ± 6.0	26.4 ± 5.8	NS
<20 years	16	5	NS
20 to 34 years	58	34	NS
≥35 years	9	6	NS
Primiparity	52	26	NS
Multiparity	31	19	NS
Unmarried	72	38	NS
Women who had a history of divorce	27	16	NS
Cigarette smoking	36	19	NS
Alcohol consumption	21	17	NS

**Table 2:** Maternal complications in the study groups. ^#^Some women had more than one complication. Data were analyzed by Fisher's exact test. NS: not significant. Attenders were found to have a lower incidence of pregnancy-induced hypertension compared with the rate of nonattenders (*P* < 0.05).

	Attenders (*n* = 83)	Nonattenders (*n* = 45)	*P* value
Pregnancy-induced hypertension	1	6	<0.05
Eclampsia	0	1	NS
Previous cesarean section	1	3	NS
Breech presentation	3	2	NS
Thyroid disease	0	2	NS
Bronchial Asthma	5	3	NS
Epilepsy	2	2	NS
Psychiatric disorder			
Schizophrenia	2	3	NS
Depression	1	2	NS
Anxiety disorder	10	1	NS
Personality disorder	14	0	<0.05
Infection			
Chlamydia trachomatis	18	5	NS
Syphilis	3	2	NS
Hepatitis B virus	0	1	NS
Hepatitis C virus	4	2	NS

**Table 3:** Pregnancy outcomes in the study groups. Data include the number of women and are presented as the mean ± standard deviation. *Four women delivered infants outside of the hospital (ambulance: two cases; on the street: one case; home: one case). ^§^One home delivery. The gestational age at delivery for nonattenders was younger than for attenders (*P* < 0.05). Significance at *P* < 0.05 was analyzed by student's *t*-test. NS: not significant. Attenders were found to have a lower incidence of preterm deliveries and emergency cesarean sections compared with the rate of nonattenders (*P* < 0.05). Attenders were found to have a higher incidence of forceps deliveries compared with the rate of nonattenders (*P* < 0.05). Significance at *P* < 0.05 was analyzed by Fisher's exact test. NS: not significant.

	Attenders (*n* = 83)	Nonattenders (*n* = 45)	*P* value
Gestational age at delivery (weeks), mean ± SD	39.4 ± 1.4	37.9 ± 2.7	<0.05
Preterm birth	3	10	<0.05
Mode of delivery			
Spontaneous delivery*	67	36	NS
Forceps delivery	8	0	<0.05
Cesarean section	8	8	NS
Planned cesarean section	5	0	NS
Emergency cesarean section	3	8	<0.05
Vaginal birth after cesarean^§^	0	1	NS
Delivery outside of a hospital	0	5	<0.05

**Table 4:** Neonatal characteristics and indications for admission to the neonatal intensive care unit. Data shown include the number of neonates and are presented as the mean ± standard deviation. *Some neonates had more than one indication for admission to a neonatal intensive care unit. Attenders were found to have a lower incidence of low birth weight compared with the rate of nonattenders (*P* < 0.05). The incidence of admission to the NICU was higher for the nonattenders than for attenders (*P* < 0.05). Neonates from nonattenders were found to have a higher incidence of preterm delivery, low birth weight, neonatal infection and asphyxia (*P* < 0.05). Significance at *P* < 0.05 was analyzed by Fisher's exact test. NS: not significant.

	Attenders (*n* = 83)	Nonattenders (*n* = 35)	*P* value
Birth weight (g), mean ± SD	2,992.5 ± 384.3	2,818.7 ± 567.9	<0.05
Low birth weight (<2,500 g)	8	13	<0.05
Macrosomia (≥4,000 g)	1	1	NS
Low Apgar score (<7) 1 min	5	5	NS
Low Apgar score (<7) 5 min	1	1	NS
Admission to neonatal intensive care unit	30	28	<0.05
Reasons for admission to a neonatal intensive care unit*			
Low birth weight (<2,500 g)	8	13	<0.05
Birth at <37 weeks' gestation	3	10	<0.05
Respiratory distress	10	10	NS
Neonatal infection	6	10	<0.05
Hyperbilirubinemia	5	5	NS
Asphyxia	1	5	<0.05
Congenital syphilis	0	2	NS
